# Phytochemical Screening and Biological Activity of *Mentha × piperita* L. and *Lavandula angustifolia* Mill. Extracts

**DOI:** 10.1155/2018/2678924

**Published:** 2018-01-10

**Authors:** Ersilia Alexa, Corina Danciu, Isidora Radulov, Diana Obistioiu, Renata Maria Sumalan, Adriana Morar, Cristina Adriana Dehelean

**Affiliations:** ^1^Banat's University of Agricultural Sciences and Veterinary Medicine, “King Michael I of Romania” from Timisoara, Calea Aradului No. 119, 300645 Timişoara, Romania; ^2^University of Medicine and Pharmacy “Victor Babes” Timisoara, Eftimie Murgu Square No. 2, 300041 Timişoara, Romania

## Abstract

The present study aimed to investigate the phytochemical composition of *Mentha* × *piperita* L. (MP) and *Lavandula angustifolia* Mill. (LA) extracts in terms of hydroxycinnamic acid (HCAs) content, in particular, caffeic (CA), p-cumaric (CU), ferulic (FE), and rosmarinic (RS) acids using LC-MS. Also, the *in vitro* antimicrobial effect against *Staphylococcus aureus* and the antiproliferative activity against two cancerous cell lines (A375 and MDA-MB-231) using the MTT assay were tested. The extracts were prepared using aromatic water which resulted from the extraction of oils from plants as extraction medium, with/without acid. The results showed that RS and FE represent the majority of HCAs compounds; the highest content of FE is found in LA (7.47 mg·g^–1^d.m.), and the maximum content of RS in MP (6.36 mg·g^–1^d.m.). Regarding the antimicrobial effect against *Staphylococcus aureus*, the two extracts showed a simulative role on the growth rate of *Staphyloccocus aureus*, but a slightly inhibitory effect (69.12%) can be attributed to the acidic environment. In terms of biological activity against MDA-MB-231 breast carcinoma cell line, and A375 human melanoma cell line, at the highest employed concentration, 150 *μ*g·mL^–1^, the tested extracts present a weak antiproliferative effect.

## 1. Introduction

The therapeutic value of medicinal plants is based on the relationship between the chemical structure of the active phytocompounds and the pharmacodynamic action they exert on the reactive elements of the body. The fact that most medicinal plants have a complex chemical composition ranging from 2-3 compounds to tens or even hundreds explains the multiple pharmacodynamic properties. *Lavandula angustifolia* Mill. (LA) and *Mentha piperita* L. (MP) are two main representatives of the Lamiaceae family widespread within the spontaneous and cultured flora all over the world being used in phytotherapy for their proven antibacterial, antioxidant, and antiproliferative action [1–4]. Treatment with LA preparations is indicated in several situations including diseases of the digestive and respiratory system, neurasthenia, overload, nervous irritability, migraines, depression, and kidney and liver diseases. LA is used in therapeutic baths to treat circulatory and rheumatic problems and also for the antipyretic action. MP is used as an adjuvant in gastrointestinal infections, digestive disorders (intestinal colic, enterocolitis, and meteorism), respiratory disorders (bronchitis, laryngitis, tracheobronchitis, and convulsive cough), pharyngitis (analgesic), nausea, nervous or mental fatigue, and mouth hygiene [3].

The polyphenolic compounds contained by the medicinal plants of the Lamiaceae family are responsible for their antioxidant capacity as well as for their antimicrobial effect. In this category of phytochemicals, an important role is played by the hydroxycinnamic acids (HCAs), in particular, caffeic (CA), p-cumaric (CU), ferulic (FE), and rosmarinic (RS) acids, compounds with a biologically active role representing one-third of the total polyphenolic compounds [5]. The medical and pharmaceutical importance of these natural compounds is due to their protective activity against the oxidative damage produced by an excess of reactive oxygen species (ROS) [1, 6]. Based on this hypothesis, the determination of the HCAs content and the correlation of their level with the antibacterial and antiproliferative activity of the LA and MP extracts represent novel aspects in elucidating the mechanisms of action of the active principles of the studied medicinal plants.

Previous researches regarding the bacteriostatic and bactericidal effect of alcoholic extracts of LA and MP highlighted the low inhibitory potential against *Staphylococcus aureus* or even the absence of bactericidal and bacteriostatic activity [7, 8]. According to our knowledge, no studies have been conducted regarding the bactericidal activity of MP and LA extracts obtained using aromatic water as extraction medium. In the process of obtaining essential oils (EOs) from LA and MP by hydrodistillation using a Clevenger device, a large amount of aromatic water is released that can be exploited in obtaining different extracts.

The present paper is intended to complete the previous research in the field aiming to study: (i) the phytochemical composition of LA and MP aromatic extracts in terms of HCAs content using LC/MS; (ii) the presence or absence of an inhibitory potential against *Staphylococcus aureus* as well as the influence of an acid extraction medium of extracts from LA and MP obtained using aromatic water; (iii) the *in vitro* antimicrobial effect of the main HCAs found in the selected extracts against *Staphylococcus aureus*, correlated with their concentration in the studied samples; and (iv) the antiproliferative activity against two cancerous cell lines (A375 and MDA-MB-231) using the MTT assay.

## 2. Materials and Methods

### 2.1. Extracts Preparation

The plants were harvested from the experimental field of local farmers from the Banat region. Species identification has been confirmed by the Department of Herbs of USAMVB, Timisoara, and a voucher sample has been retained. Fresh herbs were dried at ambient temperature and milled using a laboratory mill GM 2000 Retsch Grindomix. The extracts were obtained from 10 g of a ground plant which was subjected to extraction with 100 mL of aromatic water, obtained after hydrodistillation and separation of the essential oil. Additionally, apple vinegar was added in the extract until the solution reached a pH 4 resulting the second type of extract for testing. Extraction took place at ambient temperature, for 6 hours with agitation using a HEIDOLPH PROMAX 1020 Shaker. The extracts were stored at 4°C until the phytochemical, antimicrobial, and antiproliferative analyses were conducted. The LA and MP extracts without acid were used for the LC-MS analysis of polyphenols and MTT proliferation assay, while both acid and nonacid extracts were used for antimicrobial assay.

### 2.2. LC-MS Analysis of Polyphenols

LC-MS analysis was performed using a Shimadzu chromatograph equipped with SPD-10A UV and LC-MS 2010 detectors and EC 150/2 NUCLEODUR C18 Gravity SB 150 × 2 mm × 5 *μ*m column. Chromatographic conditions were as follows: mobile phases (A) water acidified with formic acid at pH 3 and (B) acetonitrile acidified with formic acid at pH 3, gradient program—0.01–20 min 5% B, 20.01–50 min 5–40% B, 5–55 min, 40–95% B, and 55–60 min 95% B. Solvent flow rate is 0.2 mL/min, and temperature 200°C. The monitoring wavelength was 280 nm and 320 nm. The calibration curves were performed in the range of 20–50 *μ*g·mL^−1^. The results were expressed in mg·g^−1^·dm.

The compounds were identified based on retention times (tr) and ions of interest (M/Z), as follows: CU (tr 24.4 min, M/Z 163), FE (tr 24.7 min, M/Z 193), RO (tr 28.8 min, M/Z 359), and CA (tr 21.9, M/Z 359). The limit of detection (LOD), representing the amount of compounds that could be detected with a signal to noise ratio (S/N) ≥3, was 0.5 *μ*g·mL^−1^ for CA, CU, and FE and 0.4 for RS. The limit of quantification (LOQ), representing the lowest concentration for which S/N ≥ 5, was 0.7 *μ*g·mL^−1^ for CA, CU, and FE and 0.6 *μ*g·mL^−1^ for RS. Determinations were performed in duplicate. All reagents and solvents used were analytical grade chemicals. Standards of RS, CA, and FE were purchased from Sigma-Aldrich and CU from Fluka.

### 2.3. Microbial Strain and Culture Preparation

The bacterial strain used in this study was *Staphylococcus aureus* ATCC 25923, obtained from the culture collection of the Laboratory of Microbiology in the Interdisciplinary Research Platform within Banat's “King Michael I of Romania” University of Agricultural Science and Veterinary Medicine, Timisoara. In the laboratory, the ATCC strains are maintained at −50°C. The *Staphylococcus aureus* ATCC 25923 was revived by overnight growth in Brain Heart Infusion (BHI) broth (Oxoid, CM1135), at 37°C and, subsequently, passed on BHI Agar, for 24 hours at 37°C. The strain was then diluted with saline solution 4.5‰, at an optical density (OD) of 0.5 McFarland standard (1.5 × 10^8^ UFC/mL). 1 mL of this solution was then suspended in BHI broth 1 : 30.

### 2.4. Antimicrobial Assay

Four extracts from each plant were tested on *Staphylococcus aureus*:

- Extract 1 prepared at a concentration of 100 *μ*g·mL^−1^ using aromatic water as extraction medium
- Extract 2 prepared at a concentration of 10 *μ*g·mL^−1^ using aromatic water as extraction medium
- Extract 3 prepared at a concentration of 100 *μ*g·mL^−1^ using aromatic water with pH 4
- Extract 4 prepared at a concentration of 100 *μ*g·mL^−1^ using aromatic water with pH 4

The 1 : 30 suspension of *Staphylococcus aureus* was tested using a 96 microdilution well plate. Using a Calibra digital 852 multichannel pipette, 150 *μ*L of suspension was placed in each well. The extracts were used directly and also diluted 1 : 10, placing 150 *μ*L in each well. Standards (CA, CU, FE, and RS) were also tested at concentrations of 500 and 50 *μ*g·mL^−1^, water : 96% ethyl alcohol (1 : 1, *v*/*v*). The plates were covered and left overnight at 37°C; then the OD (optical density) was measured at 590 nm using an ELISA reader (BIORAD PR 1100). Triplicate tests were performed for all samples. The *Staphylococcus aureus* suspension was used as a positive control, and the suspension mixed with water : ethyl alcohol (1 : 1) was used as a negative control.

### 2.5. MTT Proliferation Assay

A375 (human melanoma; CRL-1619™; ATCC) and MDA-MB-231 (human breast carcinoma; HTB26™; ATCC) cells were seeded onto a 96-well culture plate at a cellular density of 6000 cells/well and attached to the bottom of the well overnight. After 24 hours, 100 *μ*L of new medium containing Dulbecco's Modified Eagle's Medium (DMEM; Gibco BRL, Invitrogen, Carlsbad, CA, USA) and 50 *μ*g·mL^−1^ and 150 *μ*g·mL^−1^ of the tested extracts (dissolved in dimethyl sulfoxide (DMSO); Sigma-Aldrich Company) were added and incubated for 72 h; the medium was supplemented with 10% fetal calf serum (FCS; PromoCell, Heidelberg, Germany) and 1% penicillin/streptomycin mixture (Pen/Strep, 10,000 IU/mL; PromoCell, Heidelberg, Germany). The cells were then assayed by the addition of 10 *μ*L of 5 *μ*g·mL^−1^ MTT solution from the MTT-based *in vitro* toxicology assay kit (Tox-1; Sigma-Aldrich Company) during a 4 h contact period. The precipitated crystals were dissolved in 100 *μ*L of lysis solution provided by the manufacturer (Sigma-Aldrich Company). Finally, the reduced MTT was spectrophotometrically analyzed at 570 nm, using a microplate reader (Bio-Rad, Hercules, CA, USA). All *in vitro* experiments were carried out on two microplates in quadruplicates for each tested substance as well as controls.

### 2.6. Statistical Analysis

The mean values and standard deviations of all replicates were calculated using Excel software and Prism software package (Graph Pad Prism 4.03 for Windows). Differences between means were analyzed with a one-way ANOVA, followed by multiple comparison analysis using *t*-test. Differences were considered significant when *p* values < 0.05.

## 3. Results and Discussions

### 3.1. Polyphenols Content

**Figure 1:**
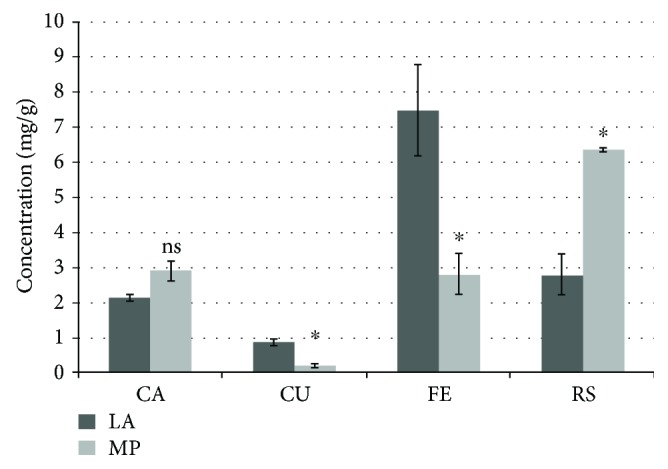
HCAs in LA and MP extracts. ^∗^The value is significantly different versus the positive control (*p* < 0.05). ns: the value is not significantly different versus the positive control (*p* > 0.05).

Results obtained fallowing the quantitative analysis of HCAs are shown in Figure 1. It can be observed that RS and FE represent the majority compounds, the highest content of FE being found in LA (7.47 mg·g^−1^·dm) and the maximum content of RS in MP (6.36 mg·g^−1^·dm). In the hydroaromatic extract of MP, the FE content was 2.79 mg·g^−1^·dm, while in the LA extract the content of RS was 2.77 mg·g^−1^·dm. The main HCAs identified in the plants belonging to the Lamiaceae family grown in Romania were FE, CU, RS, and CA. The values reported in the literature with regard to the content of HCAs vary depending on the genotype, the cultivation, and the extraction method [6]. Total HCAs (reported as rosmarinic acid/100 g dry weight) were found in quantities of 0.51 in LA 0.80 in MP [9].

RS and FE were reported as the most abundant polyphenolic constituents of LA extracts [3–5]. Sytar et al. [2] reported a FE content of 3.328 mg·g^1^·dm in LA and 0.005 mg·g^−1^·dm in MP, while other researchers identified LA cultivated in Romania that FE content comprised between 0.02–0.04 mg·g^−1^·dm [10] and 0.54 mg·g^−1^·dm in LA from Czech Republic [11] and 1.31 mg·g^−1^·dm in LA and 0.50 mg·g^−1^·dm in MP in Serbia [12].

The RS content reported by other research groups was 0.05–0.06 mg·g^−1^·dm [10] and 3.31 mg·g^−1^·dm in LA and 61.05 mg·g^−1^·dm in MP [12]. Our results are consistent with those previously reported by Vladimir-Knežević et al. [12], which revealed that the maximum RS content was found in MP, while FE was found to be the most abundant in LA.

CA and CU were detected in smaller quantities compared to FE and RS (Figure 1). The CA content was 2.39 mg·g^−1^·dm in MP and 2.13 mg·g^−1^·dm in LA, and the CU was detected at a level of 0.86 mg·g^1^·dm in LA and 0.2 mg·g^−1^·dm in MP. Comparable values with those obtained in this study have been reported by Vladimir-Knežević et al. [12] (CA 2.15 mg·g^−1^·dm in MP). Lower values in LA alcoholic extracts were obtained by Dvorackova et al. [11] (0.06–0.41 mg·g^−1^·dm). The statistical data reveals that no significant differences (ns) appear between CA content in LA and MP species, but regarding CU, FE, and RS concentration, significant differences can be observed (*p* < 0.05).

### 3.2. Antimicrobial Activity

**Figure 2:**
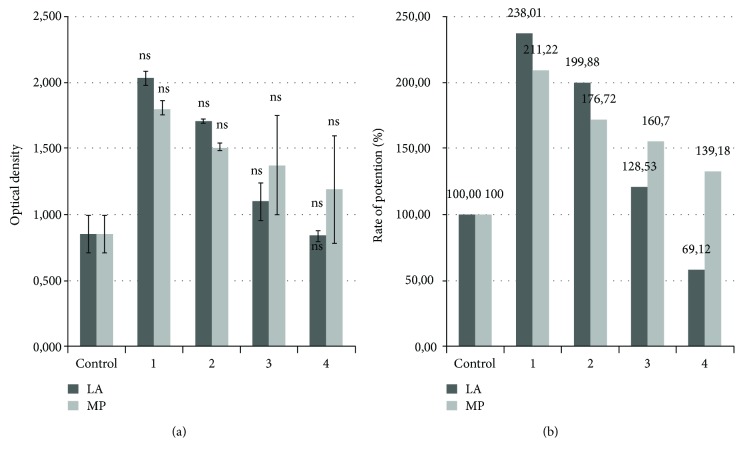
(a) Optical density (OD) of LA and MP extracts. (b) Rate of potentiation (%) of LA and MP control (BHI + *Staphylococcus aureus* inocullum; 1—extract 1 (100 *μ*g·mL^−1^), 2—extract 2 (10 *μ*g·mL^−1^), 3—extract 3 (100 *μ*g/mL^−1^, pH 4), and 4—extract 4 (10 *μ*g·mL^−1^, pH 4)).

Figure 2 presents the optical density (OD) of LA, MP extracts, and control. It can be seen that the extracts in concentrations of 10 *μ*g·mL^−1^ and 100 *μ*g·mL^−1^ do not inhibit the development of *Staphylococcus aureus*. Decreasing the concentration of LA extract 10 times (10 *μ*g·ml^−1^) leads to bacterial cell density reduction from 2.085 ± 0.051 to 1.709 ± 0.015, respectively, in the case of MP extract from 1.806 ± 0.053 to 1.511 ± 0.029. When acidic environment (extracts 3-4) was employed, a decrease in optical density (OD) was observed, the value of diluted LA extract (0.841 ± 0.21) being comparative with the control (0.855 ± 0.142), without inhibiting the microbial activity of *Staphylococcus aureus*. In the case of the MP extract, the optical density was inferior to the LA extract in experimental variants 1 and 2 (2.085 ± 0.051, 1.709 ± 0.015), but superior to the MP extract for experimental variants 3 and 4 (1.354 ± 0.378, 1.190 ± 0.406). These data reveal the fact that the obtained extracts exert a potent effect on the activity of *Staphylococcus aureus*.

The optical activity of the control sample was considered to be 100% rate of potency, and compared with this, LA extract, variants 1 and 2, exerted a potent effect of 238.01% and 199.8%, while for MP the percentage growth was 128.53%.

A slightly inhibitory effect (69.12%) can be attributed to the acidic environment in case of experimental variant 4. No significant differences (ns) were observed between the control and LA and MP extracts. Our results are in line with previous studies that reported limited bacteriostatic activity of plant extracts, including *Mentha* spices, against *Staphylococcus aureus*. Bacteriostatic activity ranged from 15.93 to 19.46% for alcoholic extracts while for aqueous extracts a lower effect was recorded [13].

At concentrations between 10 and 100 *μ*g·mL^−1^, the inhibitory capacity on *Staphylococcus aureus* was zero [13]. Moon et al. [7] described that the lavender hydrosols and aqueous extracts did not have any antibacterial activity, while Bayoub et al. [8] reported weak inhibition of LA alcoholic extract against *Staphylococcus aureus.* Compared to aqueous extracts, alcoholic extracts, especially ethanolic ones, are likely to reduce the development of Gram-positive or Gram-negative bacteria, but data on extracts obtained using aromatic water are not mentioned. Petroleum ether, chloroform, and ethyl acetate extracts were found to be more effective against *Staphylococcus aureus* than ethanol and aqueous extracts [14]. It is important to emphasize that most of the studies conducted so far refer to alcoholic or less aqueous extracts [15–18], but there are no reports regarding the antimicrobial activity of the extracts obtained using aromatic water which resulted from the extraction of oils from plants.

**Figure 3:**
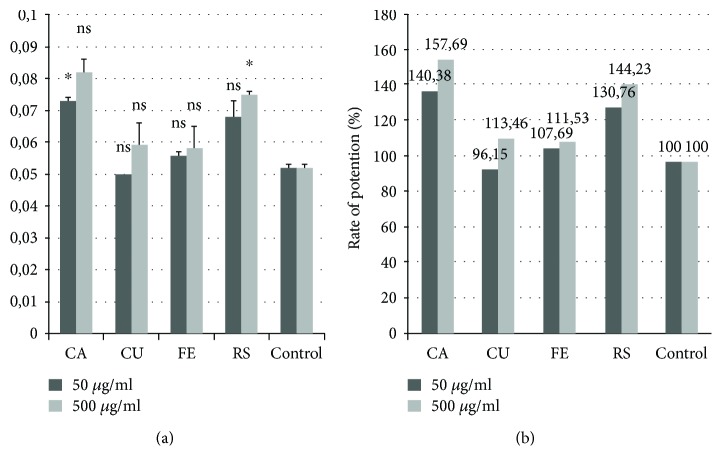
(a) Optical density (OD) of HCAs. (b) Rate of potentiation (%) of HCAs at different concentrations. ^∗^The value is significantly different versus the positive control (*p* < 0.05). ns: the value is not significantly different versus the positive control (*p* > 0.05).

Given the fact that HCs represent the main active chemical compounds in the Lamiaceae family, the effect of FE, CA, CU, and RS, prepared in concentrations of 50 *μ*g·mL^−1^ and 500 *μ*g·mL^−1^, was studied. Experimental results are shown in Figure 3.

Regarding the ability to inhibit *Staphylococcus aureus* by HCAs standards at two different concentrations (50 *μ*g·mL^−1^ and 500 *μ*g·mL^−1^), it is noted in Figure 3 that the optical density (OD) varies in the order of CU < FE < RS < CA and is stimulated by increasing the concentration of polyphenols. Expressed in percent, with the exception of CU, which at a concentration of 50 *μ*g·mL^−1^ exerts a slight inhibitory effect, a potentiation of *Staphylococcus aureus* development ranging from 111.53% to 157.69% versus control can be observed.

Previous studies have demonstrated the synergistic effects of RS with antibiotics against *Staphylococcus aureus*, but the minimum inhibitory concentration (MIC) was high [19]. Other authors highlighted the antibacterial effect of HCAs against *Staphylococcus aureus* at high concentrations: CA and CU (MIC 125–1000 *μ*g·mL^−1^) and FE (MIC 500–5000 *μ*g·mL^−1^) [20].

Considering the demonstrated potent effect of HCAs at concentrations between 50 and 500 *μ*g·mL^−1^ and the previous studies showing high MIC values, the inhibitory effect can be ensured by the synergism of active principles in the natural extracts.

### 3.3. Antiproliferative Activity

**Figure 4:**
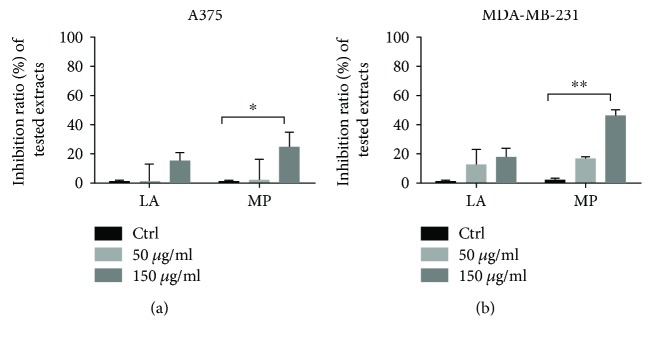
(a) Antiproliferative activity of tested extracts against A375 human melanoma cells. (b) Antiproliferative activity of tested extracts against MDA-MB-231 breast carcinoma cells. ^∗^Significant (*p* < 0.05). ^∗∗^Highly significant (*p* < 0.01).

Among the two screened cell lines, MDA-MB-231 breast carcinoma cells seemed to be more sensitive to the tested extracts in comparison to A375 human melanoma cells. In the case of this cell line, after 72 h of incubation, the effect could be detected also at the lowest concentration, namely, 50 *μ*g·mL^−1^, with the following values corresponding to the inhibition ratio: 11.53% ± 9.04% for the lavender extract (LA) and 18.57% ± 2.32% for the peppermint extract (MP). A concentration of 150 *μ*g·mL^−1^ conduced to an inhibition ratio of 16.08% ± 4.67% for the LA extract and 46.53% ± 3.04% for the MP extract. In the case of melanoma cells, the lowest tested concentrations were almost effect less. For the concentration of 150 *μ*g·mL^−1^, an inhibition ratio of 14.52% ± 3.89% could be detected for the LA extract and 25.08% ± 8.21% for the MP extract. Results are shown in Figure 4.

A recent study has analyzed the antiproliferative effect of the volatile oil obtained from *Lavandula angustifolia* Mill. against two human prostate cancer cell lines, namely, PC-3 and DU145, and concluded that the essential oil presents both *in vitro* and *in vivo* effects [21]. *In vitro* good anticancer properties have been described for the volatile oil obtained from the same species of lavender against acute lymphoblastic leukaemia cells (MOLT-4 cells) while moderate activity was depicted for breast adenocarcinoma line (MCF-7) and large cell lung cancer line (NCI-H460) [22]. Another recent paper studying the chemical composition and biological activity of five South Portugal herbs including a lavender species, *Lavandula stoechas* L. spp., *Luisieri* has been assigned to the extract obtained from this plant antiproliferative activity against HEP G2 and hepatocellular carcinoma cell line [23]. The group of Al-Ali et al. [24] published about the cytotoxic effect of methanolic extract obtained from *Mentha longifolia* L. Huds. obtained from Saudi Arabia against breast adenocarcinoma line MCF-7. Park et al. [25] have shown that menthol, one of the most important constituents of peppermint oil in terms of biological activity, was able to augment the antiproliferative effect of 1*α*,25(OH)_2_D_3_ in case of LNCaP prostate cancer cell line. Other study revealed, using the MTT assay, that ethanolic extract of *Lavandula dentata* exhibits promising cytotoxic activity with an IC50 value of 39 *μ*g·mL^−1^ against human breast adenocarcinoma (MCF-7) [26].

As mentioned in Results and Discussions, at the highest employed concentration, 150 *μ*g·mL^−1^, the tested lavender and peppermint extract present a weak antiproliferative effect against MDA-MB-231 breast carcinoma cell line and A375 human melanoma cell line.

## 4. Conclusions

As described above, the MP and LA extracts obtained with aromatic water resulted as waste in essential oil technology can represent a valuable antioxidant source represented by HCAs, especially FE and RS acids.

Biological evaluation of extracts obtained in the absence/presence of the acid environment highlights the simulative role on the growth rate of *Staphylococcus aureus*. Regarding the antimicrobial activity of standards, HCAs results indicated that these compounds have no positive influence. The antiproliferative test revealed a weak therapeutic potential against MDA-MB-231 breast carcinoma cell line and A375 human melanoma cell line.
